# Auxin and cytokinin coordinate the dormancy and outgrowth of axillary bud in strawberry runner

**DOI:** 10.1186/s12870-019-2151-x

**Published:** 2019-11-29

**Authors:** Yuting Qiu, Si Cong Guan, Chenjin Wen, Peng Li, Zhen Gao, Xu Chen

**Affiliations:** 1 College of Horticulture and Fujian Provincial Key Laboratory of Haixia Applied Plant Systems Biology, Fujian Agriculture and Forestry University, Fuzhou, Fujian China; 2 FAFU-UCR Joint Center for Horticultural Biology and Metabolomics, Haixia Institute of Science and Technology, Fujian Agriculture and Forestry University, Fuzhou, 350002 China; 3 Zibo Agriculture and Rural Affairs Service Center, Zibo, 255400 China

**Keywords:** Auxin, Cytokinin, Axillary bud, Strawberry runner

## Abstract

**Background:**

Axillary buds allow the production of either vegetative or reproductive shoots, which display a plastic developmental potential of the plant to suit the prevailing environmental changes. Strawberry represents one of many plant species which displays horizontal above-ground growth of shoot development for asexual reproduction. Two distinct runner growth patterns exist in different strawberry species: one is called sympodial type such as *Fragaria vesca*, and the other one is called monopodial type such as *Fragaria pentaphylla*. Despite the runner growth morphology of these strawberry species have been well known, the mechanisms that determine the distinct patterns have rarely been reported.

**Results:**

In this study, we used *Fragaria vesca* Hawaii-4 and *Fragaria pentaphylla* as model species, and captured the initiated dormant bud and non-dormant bud as materials to compare their transcriptome profiles and phytohormone content. Comparisons revealed that relatively higher auxin activity is present in the dormant bud and relatively higher cytokinin activity is in the non-dormant bud. Decapitation and pharmacological experiments on dormant buds showed that the reduction of auxin accumulation triggers the regeneration of vegetative shoots in dormant buds, and exogenous cytokinin application triggers cell fate turnover and generation of reproductive shoots.

**Conclusion:**

Here, we uncover a mechanism by which auxin and cytokinin coordinate the dormancy and outgrowth of axillary bud in strawberry runner. Our results suggest a contrasting behavior of auxin and cytokinin in control of axillary bud development, facilitating a preliminary understanding of shoot architecture formation in strawberry.

## Background

Axillary bud development comprises two stages: axillary meristem initiation in the leaf axil and subsequent outgrowth or dormancy [1]. The diverse patterns of axillary meristem initiation and lateral bud outgrowth cause the variation of shoot branching manners [2]. Auxin and cytokinin have been well known for a long time to coordinate a significant part of plant developmental process, including axillary meristem initiation and axillary bud outgrowth. Auxin is mainly synthesized in the shoot apex in the young leaves and is transported basipetally by auxin transporters [3, 4]. For instance, PIN-FORMEDs (PINs) are the well-known auxin efflux transporters and their polarity determines the directionality of intercellular auxin flow [5]. ATP-Binding-Cassette B (ABCB)/P-glycoprotein (PGP) members function as auxin efflux transporters, which interact and coordinate with PINs to regulate auxin efflux [6]. Additionally, AUXIN-RESISTENT1/AUX1-LIKEs (AUX1/LAXs) encodes the auxin influx transporter, which is responsive for auxin uptake within the cells [7]. Once auxin transport inhibitor 2,3,5-triiodobenzoic acid (TIBA) or N-1-naphthylphthalamic acid (NPA) was supplied to the stem, the suppression of bud outgrowth is abolished [8]. Using the DR5 and DII auxin signaling reporters, an auxin gradient is observed at the leaf axil region where axillary meristem initiates [9, 10]. Mutation of auxin efflux carrier PIN1 which is in charge of auxin gradient establishment shows remarkable axillary meristems initiation defects [9, 10]. Therefore, ectopic overproduction of auxin in the leaf axil efficiently inhibits axillary meristem initiation. On the other hand, restriction of auxin supply by inhibitors or transporter mutant results in supernumerary axillary buds [9, 10], supporting a crucial role of low auxin environment and auxin gradient maintenance for axillary meristem initiation.

Besides auxin, cytokinin is also detectable at the leaf axil of axillary bud [11]. Supplement of cytokinin restores the phenotypes of *rax* (*Regulator of Axillary Meristems*) mutant which displays a reduced number of axillary meristems due to the initiation defects [12], implying a predominant role of cytokinin during axillary meristem formation. Cytokinin metabolism is composed of biosynthesis, degradation and modification processes, requiring the activity of several key enzymes. For instance, Isopentenyltransferase (IPT) catalyses the first reaction in the biosynthesis of isoprene cytokinins, isopentenyladenine-5′-monophosphate [13]. *Lonely Guy (LOG)* genes encode cytokinin riboside 5′-monophosphate phosphoribohydrolases which are directly involved in the activation of cytokinins [14], whereas Cytokinin Oxidase (CKX), Adenine Phosphoribosyl Transferase (APT) and Uridine Diphosphate Glycosyltransferases (UGTs) convert active cytokinins to inactive conjugates [15, 16]. A lack of cytokinin biosynthesis in *ipt3,5,7* mutants results in fewer branches than wild-type (WT) plants [17, 18], and local biosynthesized cytokinin in the nodal stem promotes the outgrowth of axillary buds [19]. Cytokinin signaling is also involved in the regulation of axillary meristem formation. Cytokinin signaling is mediated by a two-component pathway, including cytokinin regulator Histidine Kinase (HK) [20], Histidine Phosphotransmitters (HPs), and separate Response Regulators (RRs) [21]. In Arabidopsis, cytokinin induces the autophosphorylation of AHK proteins, which results in the transfer of a phosphoryl group to AHP proteins. AHPs subsequently translocate from the cytosol to the nucleus, where ARRs are in turn phosphorylated to initiate transcription of cytokinin-responsive genes [22]. Plants deficient in cytokinin signaling, such as mutations of B-type *ARR* transcriptional factors all display severe defects of axillary meristem initiation [10, 18].

Additionally, auxin and cytokinin act antagonistically to coordinate axillary bud development. Auxin is able to directly inhibit cytokinin biosynthesis through an *Auxin Resistant 1 (AXR1)*-dependent auxin signaling pathway, consequently suppressing axillary bud outgrowth [23]. On the other hand, exogenous cytokinin application is able to overcome the inhibitory effect of auxin on the axillary bud activity [24]. Recent study demonstrated that cytokinin determines the transcript accumulation of auxin efflux transporter *PIN3*, *PIN4*, and *PIN7* to promote shoot branching in *arr1* mutant [25]. These accumulating evidences in the model plant *Arabidopsis* provide an appealing model that high auxin level inhibits the activity of axillary bud, while cytokinin takes the opposite effect [25]. Beside of auxin and cytokinin metabolism and signaling pathways, the activity of axillary meristem is maintained by several crucial transcriptional factors (TFs). For instance, *No Apical Meristem, Arabidopsis Transcription Activation Factor, Cup-Shaped Cotyledon* (*NAC*) family members *Cup-Shaped Cotyledon 1* (*CUC1)*, *CUC2*, and *CUC3* function redundantly to initiate axillary meristem and establish organ boundaries [26]. Interestingly, the activation of *CUC1* and *CUC2* is partly dependent on the cytokinin pathway [27]. Correspondingly, *Knotted1-Like Homeobox (KNOX)* which is expressed in a specific pattern in the shoot apical meristem, rapidly activates both cytokinin biosynthesis genes and apical meristem-localized cytokinin-responsive regulators, in turn influences axillary meristem initiation. The *KNOX* gene *Shoot Meristemless* (*STM*) functions by preventing the incorporation of cells in the meristem center into differentiating organ primordia, which is important to induce de novo meristem formation [28, 29]. *STM* also mediates the induction of cytokinin synthesis to inhibit cell differentiation, therefore stimulates the undifferentiated cells into a self-sustaining meristem [28, 29]. *Wuschel* (*WUS*) homeobox has been well known to specify stem cell identity at the shoot apical meristem, and its ectopic expression is sufficient to induce de novo shoot meristem formation [30]. Cytokinin regulator B-type *ARRs* bind to *WUS* promoter to activate its de novo expression, and stabilize *WUS* by restricting its signal in the central zone of shoot apical meristem [31–33]. Therefore, cytokinin signaling and key transcriptional factors create a regulatory circuit, fine-tuning axillary meristem initiation [1, 34, 35].

Recent researches have provided new insight into the mechanisms that control shoot architecture. Phytohormones: Strigolactone, Gibberellin (GA) and Abscisic Acid (ABA), sugar, and light have been discovered their roles in the regulation of axillary bud outgrowth. Likewise, auxin inhibits bud outgrowth by promoting the expression of strigolactone biosynthesis genes and inhibiting cytokinin biosynthesis [19, 36, 37]. Sugar and their signaling networks play a major role in the early events of bud outgrowth. Exogenous sugar supply through the petiole of plants is sufficient to induce bud outgrowth, even in the presence of auxin in the stem [38]. In addition, new evidences have demonstrated that sucrose is able to repress the auxin-induced strigolactone pathway to promote bud growth, which is largely independent of cytokinin [39]. Besides of strigolactone and sugar, the *Teosinte branched1, Cycloidea, Proliferating cell nuclear antigen factor* (*TCP*) transcription factor *BRC1* (*Branched1*) and its orthologs are specifically expressed in axillary buds, and play an important role in the response to multiple signals to control bud outgrowth [40, 41]. In pea, the expression level of *PsBRC1* can be induced by strigolactones and repressed by cytokinins and sucrose [38, 42]. Moreover, it has been reported GA regulates shoot branching through the interaction between DELLA and BRC1 [43, 44]. Furthermore, ABA might be one target of BRC1 to regulate bud outgrowth under low-light condition, as BRC1 was recently found to promote expression of *9-cis-Epoxicarotenoid Dioxygenase 3* (*NCED3*), leading to enhanced local levels of ABA under light-limiting conditions [45, 46].

Strawberry species have evolved axillary buds by producing horizontal above-ground shoots (also called runners) to achieve the asexual reproduction. However, it is still unclear whether the coordination of auxin-cytokinin in axillary bud development is applicable in strawberry. Two distinct runner growth patterns exist in different strawberry species: called sympodial and monopodial runners. Woodland strawberry *Fragaria vesca* Hawaii-4 represents one of the classical species of sympodial type runners, which alternatively develop a dormant bud (*Fv*DB) and follow with a non-dormant bud (*Fv*NDB). While *Fragaria pentaphylla* represents a typical monopodial type runner, in which consistently develops non-dormant buds (*Fp*NDB) after the first dormant bud. Further transcriptome analysis of the initiated *Fv*DB, *Fv*NDB and *Fp*NDB showed that 439 core genes were differentially expressed, and auxin and cytokinin-associated phytohormone pathways were regulated as the most significant pathways. Relatively higher auxin activity was present in *Fv*DB and relatively higher cytokinin activity was detectable in both *Fv*NDB and *Fp*NDB. Decapitation and pharmacological treatment demonstrated that reduction of auxin accumulation in *Fv*DB promoted additional bud outgrowth, whereas increasing cytokinin level in *Fv*DB altered its cell fate and stimulated the turnover of *Fv*DB to *Fv*NDB. These results provide a preliminary understanding of strawberry runner developmental pattern, and confirm the pivotal roles of auxin and cytokinin in strawberry plant growth.

## Results

### *Fragaria vesca* Hawaii-4 and *Fragaria pentaphylla* show a distinct growth manner of above-ground shoot/runner

**Fig. 1:**
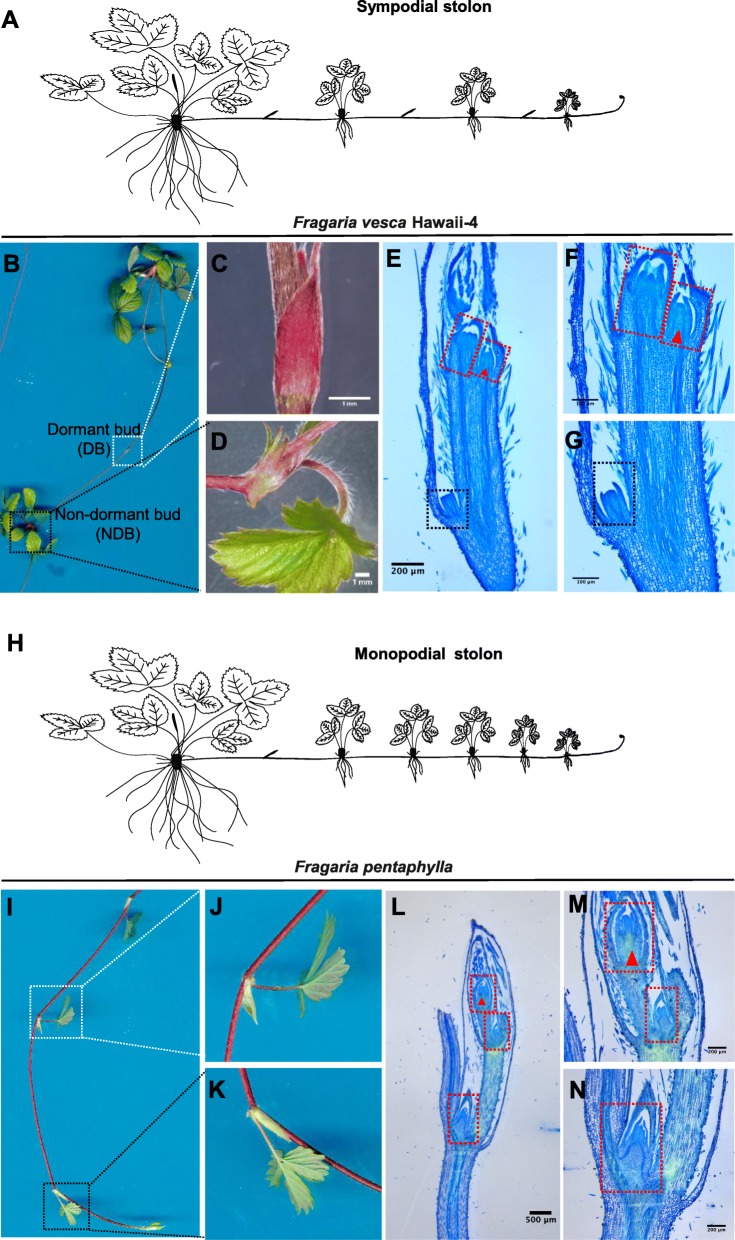
The morphological and histological structure of runner patterns in *Fragaria vesca* Hawaii-4 and *Fragaria pentaphylla*. **a** Schematic drawing of sympodial runner pattern of *Fragaria vesca* Hawaii-4. **b-d** Morphological picture of *Fragaria vesca* Hawaii-4, which produces dormant buds (C) and non-dormant buds (**d**) at varying intervals along the runner. **e**-**g** Histological images of emerged runner tips were taken by paraffin section, the dormant bud presents only axillary meristem (marked with black box), while the non-dormant bud presents both axillary meristem that will form the new runner tip (marked with red box and arrow) and shoot apical meristem (marked with red box) that will develop to form the new daughter plant. **h** Schematic drawing of monopodial runner pattern of *Fragaria pentaphylla*. **i**-**k** Except for the first bud, *Fragaria pentaphylla* produces sequential non-dormant buds. **l**-**n** The histological structure of emerged runner tip of *Fragaria pentaphylla* with active shoot apical meristem (marked with red box) and a developing axillary meristem that will form the new runner tip (marked with red box and arrow)

To understand the mechanism which determines these two types of runner growth pattern, we firstly made a classification according to the morphological property of runners in several wild and hybrid species (Additional file 6: Table S1). For instance, runners of woodland strawberry *Fragaria vesca* Hawaii-4 belong to the sympodial type (type II) (Fig. 1a), which alternatively develops a *Fv*DB and a *Fv*NDB with the property to generate a daughter plant (Fig. 1b-d). In contrast, other strawberry species such as *Fragaria pentaphylla,* show a distinct runner pattern, termed monopodial type (Fig. 1h), which forms sequential *Fp*NDBs except the first dormant bud (Fig. 1i-k).

To further dissert their morphological differences at the cellular level, we performed a longitudinal cross-section cutting. In the *Fv*DB*,* the dormant axillary meristem can be found below a non-expanded leaf axil (Fig. 1e, g). In *Fragaria vesca*, *Fv*NDB contains two meristems: one shoot apical meristem (SAM) exhibit an active cell proliferation property under an expanded leaf forms new daughter plant, and the other axillary meristem grows out to form the new runner which develops the *Fv*DB as the next node (Fig. 1e, f). In *Fragaria pentaphylla*, each *Fp*NDB contains the SAM under an expanded leaf axil with strong cell division ability to form new plantlet (Fig. 1l-n), and the axillary meristem develops to the new runner which generates the *Fp*NDB as the next node (Fig. 1l, m). Apparently, these two types of strawberry species with a distinct arrangement of bud patterns provide excellent study materials to clarify the underlying mechanisms involved in the axillary bud development in strawberry runner.

### Transcriptome analysis of the DB and NDB of *Fragaria vesca* Hawaii-4 and NDB of *Fragaria pentaphylla*

**Fig. 2:**
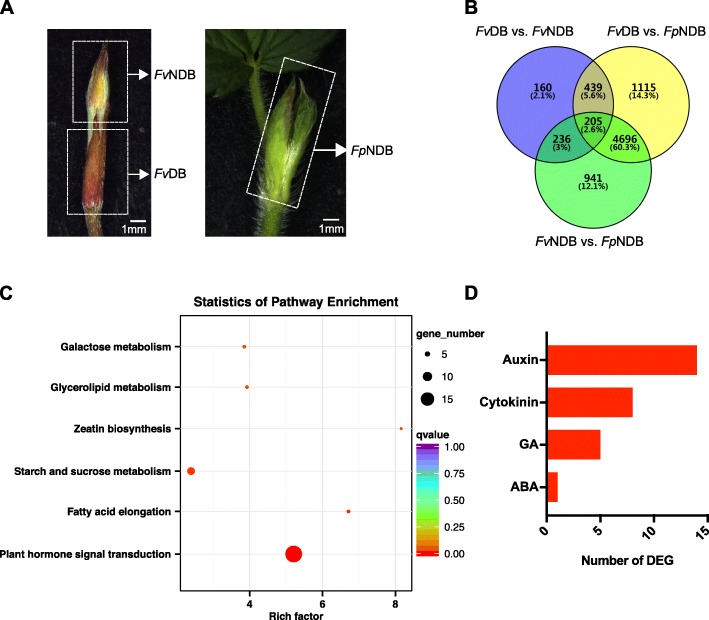
Transcriptome analysis of *Fv*DB, *Fv*NDB and *Fp*NDB. **a** The samples for RNA-seq analysis were separated from one-week old new emerged runner tips, *Fv*DB stays dormant, *Fv*NDB and *Fp*NDB are able to form new clone plant. **b** Multiple comparison of the differential expression genes (DEGs) between *Fv*DB, *Fv*NDB and *Fp*NDB. The Venn diagram shows the number of genes in *Fv*DB vs. *Fv*NDB, *Fv*DB vs. *Fp*NDB and *Fv*NDB vs. *Fp*NDB. **c** KEGG enrichment analysis of 439 DEGs in pairs of *Fv*DB and both NDB tissues in *Fragaria vesca* Hawaii-4 and *Fragaria pentaphylla.* The size of the dots indicates the number of genes enriched in this pathway and the color from red to blue indicates the q-value among 0 to 1. **d** Number of DEGs related to phytohormone pathways in the 439 core DEGs

To gain insight into the regulatory mechanisms involved in axillary bud dormancy or outgrowth in strawberry runner, RNA was isolated from the early initiated buds of *Fv*DB, *Fv*NDB and *Fp*NDB (Fig. 2a) in triplicates. The clean reads were mapped to the diploid strawberry genome (*Fragaria_vesca*_v2.0.a2) and a 74.65–93.94% mapping ratio was achieved (Additional file 6: Table S2). The high Q30 score (94.30%~ 94.49%) and appropriate GC content (above 46%) indicated a high credibility of this set of RNA sequencing (Additional file 6: Table S2). Further analysis of the overall quality of RNA-seq data with a Pearson correlation coefficient among biological replicates confirmed the high quality of RNA-seq readouts (Additional file 1: Figure S1).

Differential expression analysis was performed using the DESeq2-R package with following criteria: False Discovery Rate (FDR) < 0.01 and Log_2_FC ≥ 1. In total, 1040 Differential Expressed Genes (DEGs) were identified between *Fv*DB and *Fv*NDB (Fig. 2b, Additional file 7: Table S3). These DEGs include genes which are involved in the regulation of bud dormancy and the morphological differences between dormant and non-dormant buds. Comparison of *Fv*DB and *Fp*NDB, 6455 DEGs were identified, which offers information of bud dormancy between dormant and non-dormant buds, the morphological and species differences between *Fragaria vesca* and *Fragaria pentaphylla* (Fig. 2b, Additional file 7: Table S4). In addition, 6078 DEGs identified by comparison of *Fv*NDB and *Fp*NDB were involved in the regulation of the morphological and species differences between *Fv*NDB and *Fp*NDB (Fig. 2b, Additional file 7: Table S5). Multiple comparison analysis revealed that 160 DEGs represent exclusively for the transcriptome differences between *Fv*DB and *Fv*NDB, while 1115 DEGs represent exclusively for the transcriptome differences between *Fv*DB and *Fp*NDB (Fig. 2b). Moreover, 644 DEGs show the transcriptome differences between *Fv*DB vs. *Fv*NDB and *Fv*DB vs. *Fp*NDB. By excluding 205 DEGs related to transcriptome differences between *Fv*NDB and *Fp*NDB, 439 core DEGs might play key roles in the regulation of axillary bud dormancy or outgrowth in strawberry runner (Fig. 2b, Additional file 8: Table S6).

Analysis using the Kyoto Encyclopedia of Genes and Genomes (KEGG) enrichment categories assigned the 439 DEGs (corrected *p*-value < 0.05) in multiple pathways, in which plant hormone signal transduction was present as the most significant pathways, followed by fatty acid elongation, and starch and sucrose metabolism pathways (Fig. 2c). Phytohormones, including auxin, cytokinin, strigolactone, GA, and ABA, are major determinants of plant architecture [47]. Based on the functional annotations, these phytohormone-related DEGs were identified. Auxin and cytokinin metabolism/signaling related DEGs were grouped as the two predominant pathways (Fig. 2d). However, none of the strigolactone biosynthesis and signaling related genes was found in the 439 DEGs (Fig. 2d). The requirement of sucrose for bud outgrowth has also been reported in different plants, which is able to modulate the dynamics of bud outgrowth in a concentration-dependent manner [38, 48]. Therefore, plant hormone signal transduction and starch and sucrose metabolism are possibly involved in the regulation of axillary bud development in strawberry.

### High auxin response is present in the dormant bud

**Fig. 3:**
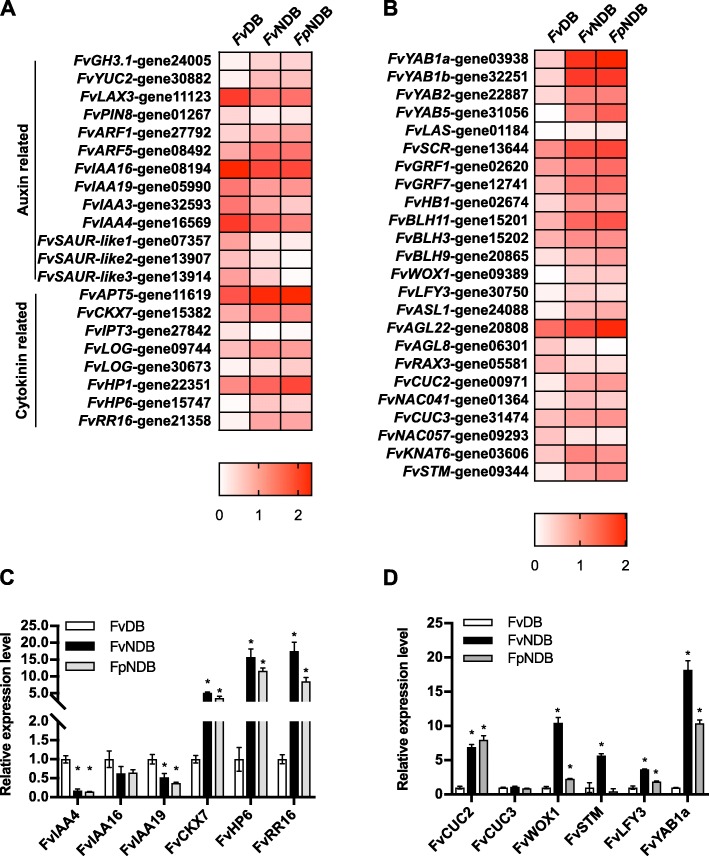
Heatmap of Auxin, cytokinin and transcription factor related genes in the 439 core DEGs. **a** Heatmap of auxin and cytokinin-related DEGs in the 439 core DEGs. **b** Heatmap of shoot apical meristem-related transcription factors in the 439 core DEGs. Heatmaps represent transformed genes by the single gradient method. White color shows the lowest value in the heatmap. in contrast, red color shows the highest value in the heatmap. **c** qRT-PCR validation of selected DEGs from (**a**), (**d**) qRT-PCR validation of selected DEGs from (B). Gene expression levels in *FvDB* were normalized to be 1. Error bars indicate the standard deviations obtained from three biological replicates. *P*-values were determined by two-tailed Student’s t-test assuming equal variances (*p < 0.05)

Based on the transcript oscillation of auxin-related DEGs, we asked whether auxin is involved in the differentiation of DB and NDB. Indole-3-acetic acid (IAA) is the major natural auxin, which is produced from indole-3-pyruvic acid by the flavin monooxygenase (YUC) proteins [49]. Heatmap showed that the expression of *FvYUC2* was upregulated in the *Fv*NDB and *Fp*NDB groups (Fig. 3a, Additional file 8: Table S7). Free auxin comprises no more than 25% of the total amount of auxin. In addition, active free auxin is converted to multiple forms of inactive auxin by acylation, esterification, methylation or glycosylation [50]. For instance, *Gretchen Hagen3 (GH3)* genes are capable to conjugate the active auxin to amino acid conjugation, thereby adjusting auxin pool for degradation/storage [51]. Transcriptome data showed that expression level of *FvGH3.1* was upregulated in *Fv*NDB and *Fp*NDB (Fig. 3a, Additional file 8: Table S7), implying that frequent conversion of free auxin to conjugated auxin is triggered in *Fv*NDB. Thus, auxin biosynthesis and conjugation pathways are relatively active in NDB than in DB.

Auxin is synthesized in the tissues with active cell division and is transported via PIN, ABCB, and AUX/LAX proteins to influence a variety of plant growth processes [52]. However, none of the plasma membrane localized auxin efflux transporter was detected in the core DEGs. Transcriptome data showed that the expression level of *FvPIN8* and auxin influx transporter *FvLAX3* was upregulated in *Fv*DB, compared to *Fv*NDB and *Fp*NDB (Fig. 3a, Additional file 8: Table S7), suggesting there was no significant auxin export from the dormant bud.

Regarding the different cells responding very differently to changes in auxin levels, plant cells require a coordination of versatile auxin signaling. Nuclear auxin signaling components consist of three protein families: The *F-box Transport Inhibitor Response 1/Auxin Signaling F-box Protein (TIR1/AFB)* auxin receptors, the *Auxin/Indole-3-Acetic Acid (AUX/IAA)* transcriptional repressors, and the *Auxin Response Factor (ARF)* transcription factors [53]. All *FvIAAs* identified in the DEGs were upregulated, but *FvARFs* were downregulated in *Fv*DB (Fig. 3a, Additional file 8: Table S7). qRT-PCR verification of these *FvIAAs* confirmed that *Fv*DB has a relatively higher expression of *FvIAAs* than *Fv*NDB and *Fp*NDB (Fig. 3c). *Small auxin up RNA* (*SAUR*) genes comprise the largest family of early auxin-responsive genes [54, 55]. In total three *FvSAURs* were found in the 439 DEGs, and all of them were upregulated in *Fv*DB (Fig. 3a, Additional file 8: Table S7). Transcripts of the Aux/IAA and SAUR families are well known to be induced rapidly in the presence of exogenous auxin [56]. The upregulation of auxin signaling-related genes in *Fv*DB suggests a possible presence of high auxin response in the dormant bud.

### High cytokinin activity is present in the NDB groups

Previous studies have stated that cytokinin acts antagonistically with auxin during axillary meristem initiation. Local biosynthesized cytokinin in the nodal stem promotes the outgrowth of axillary buds [19]. RNA-seq data showed that the expression of *FvIPT3* in *Fv*NDB and *Fp*NDB was comparably lower than in the *Fv*DB, but *FvLOG*, and *FvCKX7* were upregulated in *Fv*NDB and *Fp*NDB comparing to *Fv*DB (Fig. 3a, Additional file 8: Table S7). It has been demonstrated that the expression of LOG, which converts the cytokinin ribotides into the free-base, active forms, is important for regulating the activity of the shoot apical meristem [14, 57]. However, the level of active cytokinins is controlled via irreversible cleavage by CKXs. The expression of *CKXs* is known to be induced by cytokinins, providing a feedback mechanism to dampen cytokinin function [58]. Therefore, the upregulation of both *FvLOG* and *FvCKX7* in *Fv*NDB and *Fp*NDB groups implied that an activate cytokinin metabolism might exist in *Fv*NDB and *Fp*NDB groups to coordinate the development of the shoot apical meristem.

Beside of cytokinin metabolism, cytokinin signaling is known to regulate auxin action in the regulation of shoot branching [25], and converge in the expressional regulation of various meristem activity related transcription factors [1, 34]. In the transcriptome data, two HP genes *FvHP1*, *FvHP6* and Type-A RR gene *FvRR16* were upregulated in *Fv*NDB and *Fp*NDB, compared with *Fv*DB group (Fig. 3a, Additional file 8: Table S7). qRT-PCR verification confirmed that *Fv*NDB and *Fp*NDB contained a relatively higher expression of *FvHP6* and *FvRR16* than *Fv*DB (Fig. 3c). Normally, the AHPs are partially redundant positive regulators of cytokinin signaling [22]. But AHP6 has been identified as the cytokinin inhibitor to confine cytokinin signaling within specific tissues or layers [59]. Furthermore, *Type-A RRs*, which are transcriptionally induced in response to cytokinin by *Type-B RRs*, act as negative-feedback regulators of cytokinin signaling [60, 61]. The upregulation of *FvHP* and *Type-A RR* genes in *Fv*NDB and *Fp*NDB implies that a tightly controlled cytokinin signaling might present in *Fv*NDB and *Fp*NDB regulating the shoot meristem development.

Interestingly, RNA-seq data and qRT-PCR detection consistently showed that most of the cytokinin-regulated TFs, likewise *STM*, *WUS* and *NAC* family genes, were upregulated in *Fv*NDB and *Fp*NDB (Fig. 3b&d, Additional file 8: Table S8), further supporting the involvement of high cytokinin activity in *Fv*NDB and *Fp*NDB for the promotion of meristem development.

### qRT-PCR verification of other hormone and sucrose metablism related genes

Other phytohormones: strigolactone, GA and ABA also involve in the regulation of axillary bud outgrowth. To gain an overview of phytohormones’ functionality during runner bud development, we classified these phytohormone-related DEGs based on the functional annotations. However, none of the strigolactone biosynthesis and signaling genes was found in the 439 DEGs (Fig. 2d). Therefore, we checked the expression of several strigolactone biosynthetic and signaling genes via qRT-PCR. qRT-PCR results suggested that the expression level of strigolactone biosynthesis related genes: *FvMAX1a (More Axillary Growth 1a)*, *FvMAX1b*, *FvMAX3*, *FvMAX4,* and *FvLBO* (*Lateral Branching Oxidoreductase*) were not significantly changed when compared *Fv*DB with *Fv*NDB, but significantly downregulated in the *Fp*NDB (Additional file 4: Figure S4A). In addition, strigolactone signaling gene *FvD14* (*Dwarf14*) was downregulated in the *Fv*NDB, but upregulated in the *Fp*NDB, compared to *Fv*DB (Additional file 4: Figure S4A). Moreover, *FvBRC1* was significantly downregulated in the *Fp*NDB (Additional file 4: Figure S4A).

Five GA biosynthesis and signaling genes, and ABA receptor *FvPYL4* (*Pyrabactin Resistance Like 4*) were found in the DEGs (Fig. 2d and Additional file 4: Figure S4B). Regarding to the transcriptome data, *FvGA20OX1 (GA20-oxidase1)*, GA signaling regulator *FvSLY2* (*Sleepy2*) and ABA receptor *FvPYL4* exhibited lower expression in *Fv*DB than *Fv*NDB and *Fp*NDB, while relatively higher expression of GA biosynthesis genes *FvKAO2(ent-Kaurenoic Acid Oxidase 2)*, *FvGA20OX3* and *FvGA20OX4* were found in *Fv*DB (Additional file 4: Figure S4B). qRT-PCR results confirmed these transcriptome results, except the expression level of *FvSLY2,* it was not significantly altered between *Fv*DB and *Fp*NDB (Additional file 4: Figure S4C). The expression of four ABA biosynthesis gene *FvNCEDs* was also verified through qRT-PCR. The results indicated that only *FvNCED1–1* was significant upregulated in *Fv*DB (Additional file 4: Figure S4C).

Sugar has been considered as one key regulator in the early events of bud outgrowth. In the KEGG enrichment, starch and sucrose metabolism was also enriched as one important pathway. Therefore, we verified the expression of several sugar metabolism and signaling genes. qRT-PCR results confirmed the significant upregulation of *FvTPS11* (*Trehalose-6-Phosphate Synthase 11*), *FvBFRUCT1* (*Beta-Fructofuranosidase 1*), *FvAGAL2*(*Alpha-Galactosidase 2*) in *Fv*DB, while the transcripts level of *FvBXL4* (*beta-D-xylosidase 4*) was upregulated in *Fv*NDB and *Fp*NDB (Additional file 4: Figure S4B&C). *TPS11* is strongly repressed by sucrose [62]. Therefore, a high level of sucrose might exhibit in *Fv*NDB and *Fp*NDB.

### Measure of phytohormone content in the *Fv*DB, *Fv*NDB and *Fp*NDB

To understand the biological relevance between phytohormone content and strawberry runner development, we measured free auxin, cytokinin and ABA content in the *Fv*DB, *Fv*NDB and *Fp*NDB by HPLC (Addition file 2: Figure S2). Phytohormone measurement data showed that *Fv*DB has a significant higher level of ABA than *Fv*NDB. It has been reported that ABA controls seed dormancy in different plants [63]. Therefore, the high level of ABA might contribute to the bud dormancy in *Fv*DB. Interestingly, the ABA content in the *Fp*NDB is even more than *Fv*DB, indicating other regulators might act an antagonistic role to ABA in the *Fp*NDB.

**Fig. 4:**
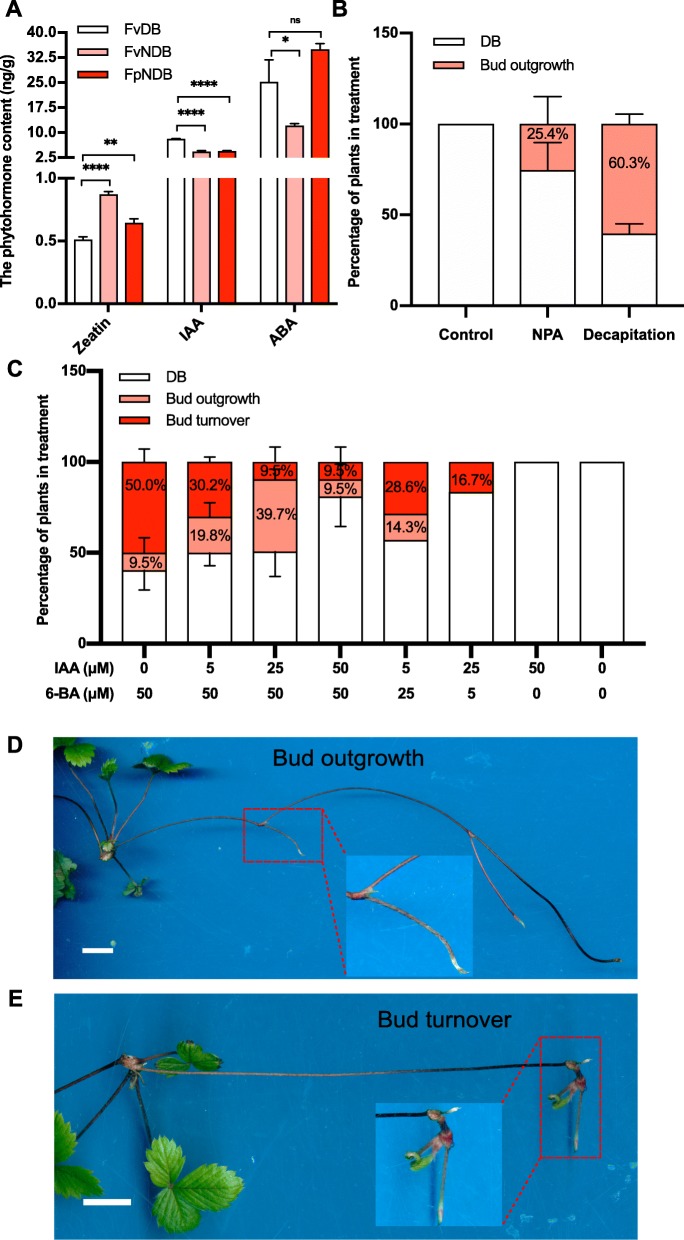
Cytokinin treatment is capable to change the *Fv*DB identity. **a** The IAA, zeatin and ABA level were detected in *Fv*DB, *Fv*NDB and *Fp*NDB by HPLC. The data were obtained from three biological replicates. P-values were determined by two-tailed Student’s t-test (*p < 0.05, **p < 0.01, ****p < 0.0001). **b** Statistical analysis of the bud outgrowth frequency in *Fv*DB upon NPA treatment (100 μM for 14 days) or decapitation. The data were obtained from three biological replicates, (*N* = 20, 19, 20 for control, NPA, decapitation, respectively). **c** Statistical analysis of the bud outgrowth and turnover frequency in *Fv*DB upon 6-BA and IAA co-treatment for 16 days (concentrations were indicated in the chart). The data were obtained from three biological replicates (From left to right, N = 20, 20, 20, 20, 21, 18, 15, 15, respectively). **d** NPA or decapitation promotes bud outgrowth of *Fv*DB. Red box highlighted the new runner that was growing from the dormant-bud, and the two-times enlarged picture was shown. **e** Cytokinin treatment is able to switch the *Fv*DB to *Fv*NDB like identity. Red box highlights the turn-over bud which was shifted from dormant to non-dormant identity, and the two-times enlarged picture was shown

In addition, *Fv*DB contained two-fold higher IAA, but 1.6-fold lower *trans-zeatin* (the most common form of natural cytokinin) than *Fv*NDB (Fig. 4a & Additional file 3: Figure S3). *Fp*NDB has comparable IAA level as *Fv*NDB. The *trans-zeatin* level of *Fp*NDB was significantly lower than *Fv*NDB but higher than *Fv*DB (Fig. 4a). Clearly, a high auxin/cytokinin ratio exists in the *Fv*DB, while *Fv*NDB and *Fp*NDB has a higher cytokinin/auxin ratio. Considering the growth pattern and corresponding phytohormone level of *Fv*DB, *Fv*NDB and *Fp*NDB, it strongly suggested that a high auxin/cytokinin ratio might keep the dormancy of the axillary buds, while a high cytokinin/auxin ratio stimulates the development of the axillary buds. The different auxin/cytokinin ratio might play a crucial role in the formation of distinct runner pattern between *Fragaria vesca* and *Fragaria pentaphylla*.

### Auxin keeps the dormancy of the axillary buds, but cytokinin releases and promotes the axillary meristem development

To verify auxin and cytokinin’s effect on the *Fv*DBs, we decapitated the apical *Fv*NDBs, or applied auxin transport inhibitor NPA, to block the auxin transportation from the apical bud to the main stem. In total, 25.4 ± 15.1% NPA-treated *Fv*DBs and 60.3 ± 5.5% decapitated *Fv*DBs result in bud outgrowth (dormancy buds released from dormancy to generate new runners) (Fig. 4b and d). We further applied exogenous auxin (IAA) and cytokinin (6-BA) on the *Fv*DBs. With 50 μM 6-BA treatment, 9.5 ± 8.2% *Fv*DBs result in bud outgrowth (Fig. 4c). Moreover, 50.0 ± 7.1% *Fv*DBs result in bud turnover (dormancy buds released from dormancy and changed identity to NDBs, which are able to form new ramets) (Fig. 4c & e). However, no significant changes happened to the *Fv*DBs with 50 μM IAA treatment. We next sprayed IAA and 6-BA on *Fv*DB in dose combination. Five micrometre 6-BA plus 25 μM IAA resulted in 16.7% of bud turnover. Twenty five micrometre 6-BA plus 5 μM IAA increased the frequency of axillary bud release to be 28.6% bud turnover and 14.3% bud outgrowth (Fig. 4c). In order to distinguish the promotion of bud turnover is caused by the increased cytokinin level or decreased auxin level, we co-supplied 50 μM 6-BA with dose concentration of IAA (0–50 μM). Compared with 50 μM 6-BA treatment alone, the increased concentration of co-treated IAA obviously decreased the frequency of bud turnover (Fig. 4c). Thus, auxin suppresses the activity of cytokinin on the regulation of axillary meristem development.

**Fig. 5:**
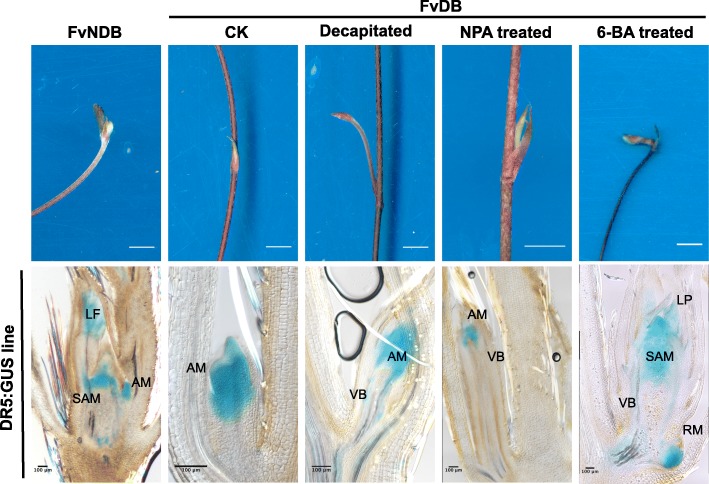
Morphology and auxin distribution differences between *Fv*NDB and treated *Fv*DBs. Upper row scanning pictures show the morphology differences of *Fv*NDB, untreated *Fv*DB, decapitated *Fv*DB, NPA treated *Fv*DB and 6-BA treated *Fv*DB. After 2 days decapitation or 14 days 100 μM NPA treatment, the dormant bud grew out as new runner. After 14 days 50 μM 6-BA treatment, *Fv*DB changed to *Fv*NDB like identity. Scale bar = 1 cm. Lower row DR5:GUS straining pictures show the auxin distribution in the related upper figures, scale bar = 100 μm. SAM: Shoot apical meristem; AM: Axillary meristem; VB: Vascular bundles; LF: Leaf; LP: Leaf primordium; RM: Root meristem

In order to compare the change of auxin responses under different treatment, we used the DR5:GUS transgenic strawberry plant (*Fragaria vesca* Hawaii-4 background). DR5 activity was detectable in both the *Fv*NDBs and the *Fv*DBs. In the *Fv*NDB, DR5-labelled auxin level was mainly accumulated in the shoot apical meristem, as well as in the non-expanded leave and the below axillary meristem (Fig. 5 & Additional file 5: Figure S5). In the *Fv*DB, high auxin level was found in the whole axillary meristem (Fig. 5 & Additional file 5: Figure S5). After decapitation or NPA treatment, DR5-labelled auxin level in the axillary meristem was significantly decreased. While GUS staining was detected in the vascular bundles, suggesting a consistent auxin depletion via auxin polar transport in the vascular bundles occurs during axillary bud outgrowth. After 6-BA treatment, auxin response in those identity altered *Fv*DB displayed a similar distribution pattern as the untreated *Fv*NDB. Moreover, a strong auxin response was found in the new formed root primordium at the basal of the altered *Fv*DB (Fig. 5 & Additional file 5: Figure S5).

### qRT-PCR verification of transcript changes after decapitation and hormone treatment

**Fig. 6:**
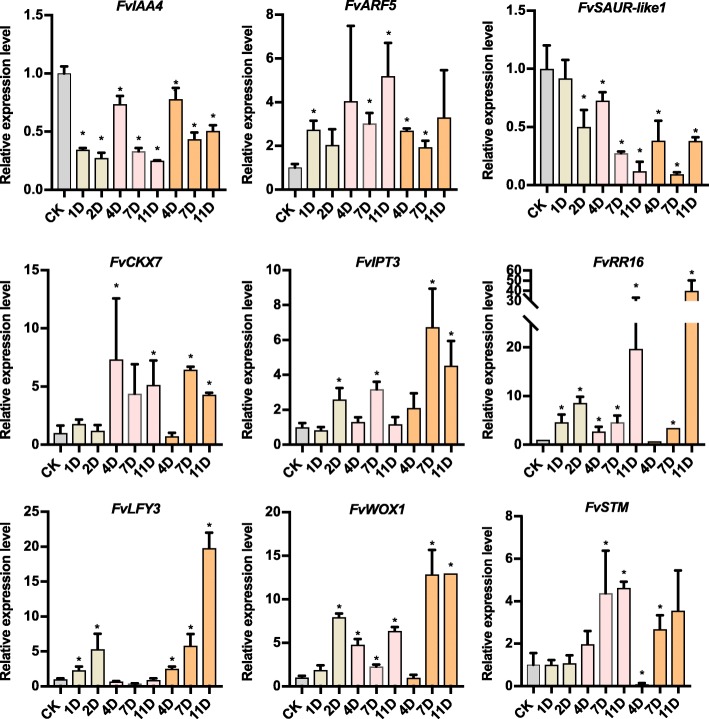
The relative expression level of nine selected DEG genes. qRT-PCR analysis of auxin synthesis and metabolic-related genes (*FvIAA4*, *FvARF5* and *FvSAUR-like1*), cytokinin synthesis and metabolic-related genes (*FvCKX7*, *FvIPT3* and *FvRR16*), and shoot apical meristem-related transcriptional factors (*FvWOX3*, *FvSTM* and *FvLFY3*). Error bar indicates the standard deviation obtained from three biological replicates. P-values were determined by two-tailed Student’s t-test (*p < 0.05)

At the transcriptional level, the expression of *FvARF5* was significantly upregulated after decapitation or hormone treatment, while *FvIAA4*, and *FvSAUR-like1* were significantly downregulated. Taken together with the decreased auxin concentration in the *Fv*DBs (Fig. 4 a, Figs. 5 and 6), these data demonstrated that reduction of the auxin level in the *Fv*DB can release the dormant axillary buds, leading to the outgrowth of new runners in strawberry. On the other hand, cytokinin signaling/metabolism genes, such as *FvCKX7*, *FvIPT3* and *FvRR16* were pronouncedly upregulated in *Fv*DB after decapitation, upon NPA and 6-BA treatment, compared to the non-treated group (Fig. 6). Correspondingly, plenty of shoot apical meristem-related TFs were stimulated in the *Fv*NDB, correlated with the active cytokinin signaling/metabolism (Fig. 3b). Consistently, qRT-PCR analysis showed that a remarkable increase of *FvLFY3*, *FvSTM* and *FvWOX1* transcript was detectable after NPA treatment or 6-BA treatment (Fig. 6). Therefore, low auxin level or high cytokinin is sufficient to stimulate the activity of apical meristem-responsive genes, which were probably involved in the activation of axillary meristem development. All these results indicated that auxin and cytokinin act antagonistically to regulate the growth pattern of strawberry axillary buds, by which auxin keeps the dormancy of axillary bud and cytokinin changes the cell fate of the dormant axillary meristem.

## Discussion

Shoot architecture is highly varied among different plant species, which consists of the spatial arrangement of stems, leaves and the reproduction of secondary shoots [64, 65]. Different than other plants with an identical shoot architecture, two types of runner architectures: sympodial and monopodial, exist in different strawberry species. In the runner of *Fragaria vesca*, *Fv*DB and *Fv*NDB are alternatively formed, while in *Fragaria pentaphylla*, *Fp*NDBs are sequentially generated along the runner. The different architectural types in *Fragaria vesca* and *Fragaria pentaphylla* offer a good model to understand how different strawberry species control the fate of axillary meristems to adapt different natural environment conditions.

Transcriptome analysis of *Fv*DB, *Fv*NDB and *Fp*NDB demonstrated that plant hormone signal transduction might play an important role in the regulation of axillary bud development in strawberry runner (Fig. 2c). Auxin and cytokinin metabolism/signaling related DEGs were grouped as the two predominant pathways (Fig. 2d). Phytohormone measurement and pharmacological experiments have firmly proved that high auxin accumulation inhibits the outgrowth of axillary bud. In contrast, suppression of auxin activity by high level of cytokinin promotes the outgrowth of axillary bud. Various studies have revealed the antagonistic roles of the plant hormones auxin and cytokinin during shoot architecture formation [18, 25, 40]. According to the lasting model, auxin is produced in the shoot apical and transported basipetally along the shoot which is important for the establishment of competition between branches [41]. Auxin transport in the main stem blocked auxin export from the axillary buds, leading to bud dormancy [47, 65]. Contrarily, cytokinin can be transported into the buds to promote bud release. Recent evidences have proposed that cytokinin might regulate bud outgrowth through the control of auxin transport [25]. Decapitation, NPA or cytokinin treatment is able to remove the inhibition of auxin export in the axillary buds, resulting in the release of dormant buds (Fig. 5 & Additional file 5: Figure S5). Therefore, Auxin and cytokinin play antagonistic roles in the regulation of axillary bud dormancy or outgrowth during runner development in strawberry.

Beside of auxin and cytokinin, strigolactone is also required to regulate axillary bud growth. On the one hand, strigolactone acts antagonistically with auxin by enhancing PIN1 internalization to reduce auxin transport in the stem [66, 67]. On the other hand, external application of strigolactone inhibited both decapitation or cytokinin-induced axillary shoot length [68, 69]. However, strigolactone biosynthesis and signaling related genes were not found in the 439 core DEGs of our RNA-seq data (Additional file 8: Table S6). qPCR verification also showed that the expression level of several *FvMAX* genes and *FvBRC1* was not significantly altered when we compared the *Fv*DB and the *Fv*NDB (Additional file 4: Figure S4A). Interestingly, previous study has indicated that a long-distance transport of strigolactone happens from the root to the shoot [70]. Based on the possible requirement of nutrient availability for strawberry DB/NDB pattern formation [71], strigolactone-regulatory pathway might integrate the long-distance transport of nutrient through source to sink to mediate the development of strawberry runner internodes. Thus, it is possible to obtain the differentially expressed strigolactone-related genes via internodes. Altogether, the axillary bud growth pattern among different strawberry species is not sufficiently explained by the sole auxin/cytokinin model, which requires the integration of a far more complex system.

KEGG pathway enrichment suggests that sucrose/starch metabolism presents as another possible regulatory machinery involved in strawberry runner pattern formation (Fig. 2c). In our transcriptome data, sugar metabolism and signaling gene *FvTPS11* showed a higher expression level in the *Fv*DB. As *TPS11* is repressed by sucrose [62], suggesting more sugar content in *Fv*NDB and *Fp*NDB. In Arabidopsis, AtTPS1 controls the production of Tre6P (Trehalose-6-phosphate), an important sugar signaling metabolite [72]. Recent studies have shown that sugars not only play a nutritional role, but also serve as an important signaling mediator for bud release [38]. For instance, Tre6P mediated signaling might regulate the early events of bud release in pea [73]. Importantly, sucrose might be able to repress the auxin-induced strigolactone pathway independent of cytokinin to promote bud growth [39]. Therefore, further investigations to reveal the interaction between sugar and hormonal signaling could add the missing knowledge during strawberry bud development.

Beside the release of *Fv*DB from dormancy, our studies also showed that high level of cytokinin treatment changed the cell fate of *Fv*DB (Fig. 4c&e). Our transcriptome data indicated that plenty of shoot apical meristem-related transcriptional factors were stimulated in the *Fv*NDB (Fig. 3b, Additional file 8: Table S8), suggesting an underlying role of the key transcriptional factors in the control of strawberry bud development. In the framework of the *Arabidopsis* shoot apical meristem formation, *WUSCHEL-*mediated transcriptional network, auxin and cytokinin signalings define a universal feedback system to achieve the stem cell number and proliferation in the meristem zone [30, 74]. Therefore, a dynamic local transcriptional and global hormone signals are both essential for meristem development. Our study also found that cytokinin application increases the transcripts of meristem-related transcriptional factors, such as *FvLFY3*, *FvSTM* and *FvWOX1,* in the treated *Fv*DBs (Fig. 6), in line with the previous conclusion that cytokinin activates *AtSTM* and *AtWOX3* to maintain the activity of *Arabidopsis* shoot apical meristem [75, 76]. Therefore, identification of key transcriptional factors that determine the strawberry bud development would deliver more comprehensive knowledge of the architecture establishment in the different strawberry species.

## Conclusion

Strawberry runner is considered as a horizontal shoot that runs above the ground and continually generates new daughter plants during its elongation. The sympodial type *Fragaria vesca* Hawaii-4 and the monopodial type *Fragaria pentaphylla* were used as model species to understand the regulatory mechanism of strawberry runner bud development. We compared the global transcriptome data between *Fv*DB, *Fv*NDB and *Fp*NDB, and we found that auxin and cytokinin served as essential phytohormones to coordinate axillary bud activity. The following pharmacological and physiological experiments all support the conclusion that high auxin level restricts the outgrowth of axillary bud, but high cytokinin level triggers the burst of non-dormant bud.

## Methods

### Plant materials and growth condition

*Fragaria vesca* Hawaii-4 (https://npgsweb.ars-grin.gov/gringlobal/taxonomydetail.aspx?id=403289) and *Fragaria pentaphylla* (https://npgsweb.ars-grin.gov/gringlobal/taxonomydetail.aspx?id=317939) species were provided by Yuntao Zhang. The strawberry transgenic DR5:GUS plant (*Fragaria vesca* Hawaii-4 Background) was obtained from Chunyin Kang’s lab. All the strawberry plants were grown in the PINDSTRUP and vermiculite 4:1 substrate in a 15 × 15 × 15 cm^3^ pot, and placed in the greenhouse with a 16-h light/8-h dark photoperiod, 23 °C, and 60% humidity.

### Histological studies and microscopic analyses

*Fv*DB, *Fv*NDB, and *Fp*NDB tissues were collected, and fixed in formalin-acetic acid-alcohol (FAA) overnight. Paraffin section preparation was performed as described by Hollender et al. [77]. Samples were sliced into 15 μm sections by Leica RM2255. After stained by Toluidine blue, pictures were captured under Nikon Ni-U DIC microscopy.

### Endogenous hormone measurement

The runner tissues of *Fv*DB, *Fv*NDB and *Fp*NDB were ground to powder in liquid nitrogen for hormone extraction and measurement. Every 100 mg sample powders were used for IAA, Cytokinin, ABA extractions with three biological replicates. Samples were dissolved in 900 μL methanol (70%, v/v) and 100 μL internal standard (^13^C_6_-IAA 100 ng/mL, ^15^N_4_-trans Zeatin 100 ng/mL, ^2^H_6_-ABA 100 ng/mL). After half-hour ultrasonic, samples were kept overnight at − 20 °C. The next day, the samples were taken out and sonicated for 30 mins, and extracts were centrifuged in 4 °C for 10 min at 14,000×g. The first supernatant was collected. And then, added 500 μL methanol (70%, v/v) to the precipitate, sonicated for 30 min, and centrifuged in 4 °C for 10 min at 14,000×g. The second supernatant was collected. The supernatants were concentrated in a SpeedVac (Thermo Fisher) to 300 μL. After that, 700 μL 1% formic acid(v/v) was added to supernatant and vortexed for 1 min, the supernatant was kept at − 20 °C for 3 h. Solid-phase extraction (SPE, Oasis MCX extraction cartridge, 60 mg 3 mL) was activated by 2 mL 70% methanol, 2 mL 0.1 M HCl, 2 mL 1% formic acid. The samples were loaded to SPE. Interference was flushed with 2 mL 1% formic acid, and eluted IAA and ABA with 2 mL 70% methanol. Then, interference was flushed with 2 mL 70% methanol, and eluted *trans-Zeatin* with 2 mL 5% ammonium hydroxide. Next, these fractions were concentrated to dryness, 200 μL methanol (70%, v/v) was added and hormones in these fractions were analyzed. The hormone measurement was performed using HPLC as described by Ma et al. [78].

### Exogenous hormone and decapitation treatment

The one-week old new emerged runner tip (with *Fv*DB and *Fv*NDB just separated morphologically, as Fig. 2a-b) were collected for hormone treatment. Runners were treated with, 50 μM 6-BA, 50 μM 6-BA&5 μM IAA, 50 μM 6-BA&25 μM IAA, 50 μM 6-BA&50 μM IAA, 25 μM 6-BA&5 μM IAA, 5 μM 6-BA&25 μM IAA, 50 μM IAA and DMSO as control for 16 days. Each treatment was executed with three replicates, *N* = 20, 20, 20, 20, 21, 18, 15, 15, respectively.

For NPA treatment, 100 μM NPA were applied to runners for 16 days. DMSO was applied to runners as control for 16 days. For decapitation treatment, the runner tips (*Fv*NDB) were cut, pictures were captured on 2 days after decapitation. Each treatment was executed with three replicates, N = 20,19,20 for control, NPA, and decapitation, respectively.

### RNA-seq, annotation, DEG, GO and KEGG pathway enrichment analyses

Total RNA was extracted from runner tissues of *Fv*DB, *Fv*NDB and *Fp*NDB using RNA prep Pure Plant Kit (Tiangen). The quality and purity of total RNA were evaluated by Nanodrop 2000 (Thermo Fisher Scientific Inc., Waltham, USA). Total RNA samples with a quality value greater than RNA integrity number (RIN) = 8 were sequenced on an Illumina Hi-seq 2500 platform, and 150 bp paired-end reads (PE150) were generated by following the manufacturer’s recommendations.

Raw data (raw reads) of fastq format were firstly processed through in-house perl scripts. The clean reads were then mapped to the diploid strawberry genome *Fragaria_vesca*_v2.0.a2 using Tophat with the default parameters. Differential expression analysis of two groups was performed using the DESeq R package. DEGs between two samples were identified according to the following criteria: FDR < 0.01 and |log 2 (fold change) | ≥ 1. Gene Ontology (GO) enrichment analysis of the DEGs was implemented by the GOseq R packages based on Wallenius non-central hyper-geometric distribution [79], which can adjust for gene length bias in DEGs. KOBAS software was used to test the statistical enrichment of differential expression genes in KEGG pathways [80].

All of the *Fragaria vesca* TFs were identified with references as described by Li et al. [81], and the hormone genes were identified by BLAST against *Fragaria vesca* Gene Models (Hybrid V2) using *Arabidopsis* protein sequences as query [82]. Heatmaps were created by Graphpad prism 7 with FPKM transformed by y = log_10_(y + 1) method.

### qRT-PCR assay

First-strand cDNA was synthesized from total RNA using the PrimeScript RT reagent kit with gDNA Eraser (TaKaRa, Japan) according to the manufacturer’s instructions. qRT-PCR was performed using SYBR Green PCR master mix (TransGen, China) on a Bio-Rad Real-Time system (Bio-Rad, Hercules, USA). *FvACTIN* was used as an internal control [83]. Primers designed from the conserved region of each cDNA were used for qRT-PCR analyses (Additional file 9: Table S9). Relative expression levels were calculated using the 2 ^−ΔΔCT^ method.

### GUS staining

The DR5:GUS transgenic *F. vesca* Hawaii-4 plants were used for hormone treatment. After treatment, samples were embedded in 5% agarose and sliced into 60 μm sections under Leica VT1000. Samples were incubated in X-Gluc solution (final concentration 1 mg/mL X-Gluc, dissolved in DMF) at 37 °C for 3 h, bleaching with 75% ethanol, and then pictures were captured under Nikon NI-U DIC microscopy.

## Supplementary information

**Additional file 1: Figure S1.** Correlation analysis. The value r 2 close to 1 means a stronger correlation between two samples.

**Additional file 2: Figure S2.** Volcano plot of different expression analysis. (A) volcano plot of DEGs between *Fv*DB and *Fv*NDB. (B) volcano plot of DEGs between *Fv*DB and *Fp*NDB. (C) volcano plot of DEGs between *Fv*NDB and *Fp*NDB. Differential expression analysis was performed using the DESeq2-R package with following criteria: FDR < 0.01 and Log_2_FC ≥ 1.

**Additional file 3: Figure S3.** IAA and Zeatin content were detected by HPLC-MS. The representative chromatograms showed LC separation and MS detection with ESI mode of 13 C 6 -IAA (A) and 15 N 4 -trans Zeatin (C). The standard curves of 13 C 6 -IAA (B) and 15 N 4 -trans Zeatin (D) were made by 5 concentration gradients.

**Additional file 4: Figure S4.** (A) The relative expression level of Strigolactone related genes. Error bar indicates the standard deviation obtained from three biological replicates. *P*-values were determined by two-tailed Student’s t-test *p < 0.05. (B) Heatmap of GA, ABA and sugar metabolism and signaling related genes in the 439 core DEGs. Heatmaps represent transformed genes by the single gradient method. White color shows the lowest value in the heatmap. in contrast, red color shows the highest value in the heatmap. (C) The relative expression level of GA, ABA and sugar metabolism and signaling related genes. Error bar indicates the standard deviation obtained from three biological replicates. P-values were determined by two-tailed Student’s t-test *p < 0.05.

**Additional file 5: Figure S5.** Replicates of DR5:GUS straining pictures. 6-BA treated FvDB have two different development types after release from dormancy. One is bud turnover, another is bud outgrowth. SAM: Shoot apical meristem; AM: Axillary meristem; VB: Vascular bundles; LF: Leaf; LP: Leaf primordium; RM: Root meristem

**Additional file 6: Table S1.** Runner pattern in different species of *Fragaria.* **Table S2.** Summary of transcriptome sequencing

**Additional file 7: Table S3** DEGs identified between *Fv*DB and *Fv*NDB. **Table S4** DEGs identified between *Fv*DB and *Fp*NDB. **Table S5.** DEGs identified between *Fv*NDB and *Fp*NDB

**Additional file 8: Table S6.** Four hundred thirty nine core DEGs response for the transcriptome differences between *Fv*DB and both NDB tissues in *Fragaria vesca* Hawaii-4 and *Fragaria pentaphylla*. **Table S7.** The expression data of phytohormone-related genes corresponding to Fig. 3a. **Table S8.** The expression data of transcription factors corresponding to Fig. 3b

**Additional file 9: Table S9.** The primers list used in this study.

## Data Availability

*Fragaria pentaphylla* The datasets used and/or analyzed during the current study are available from the corresponding author on reasonable request.

## Abbreviations

6-BA: 6-Benzylaminopurine
ABA: Abscisic Acid
ABCB: ATP-Binding-Cassette B
AGAL2: Alpha-Galactosidase 2
AHP: Arabidopsis Histidine Phosphotransmitter
APT: Adenine Phosphoribosyl Transferase
ARF: Auxin Response Factor
ARR: Arabidopsis Response Regulator
AUX/IAA: Auxin/Indole-3-Acetic Acid
AUX1/LAX: AUXIN-RESISTENT1/AUX1-LIKE
AXR1: Auxin Resistant 1
BFRUCT1: Beta-Fructofuranosidase 1
BRC1: BRANCHED1
BXL4: Beta-D-xylosidase 4
CKX: Cytokinin Oxidase
D14: Dwarf14
DB: Dormant bud
DEG: Differential Expressed Gene
DMF: Dimethylfumarate
FAA: Formalin-acetic acid-alcohol
FDR: False Discovery Rate
Fp: Fragaria pentaphylla
FPKM: Fragments per Kilobase Million
Fv: *Fragaria vesca*
GA: Gibberellin
GA20OX: GA20-oxidase
GH3: Gretchen Hagen3
GO: Gene Ontology
HK: Histidine Kinase
HP: Histidine Phosphotransmitter
IAA: Indole-3-Acetic Acid
IPT: Isopentenyltransferase
KAO2: ent-Kaurenoic Acid Oxidase2
KEGG: Kyoto Encyclopedia of Genes and Genomes
KNOX: Knotted1-Like Homeobox
LBO: Lateral Branching Oxidoreductase
LFY: LEAFY
LOG: Lonely Guy
MAX: More Axillary Growth
NAC: No Apical Meristem, Arabidopsis Transcription Activation Factor, Cup-Shaped Cotyledon
NCED3: 9-cis-Epoxicarotenoid Dioxygenase 3
NDB: non-dormant bud
NPA: N-1-naphthylphthalamic acid
PGP: P-glycoprotein
PIN: PIN-Formed
PYL4: Pyrabactin Resistance Like 4
RAX: Regulator of Axillary Meristems
RIN: RNA integrity number
RR: Response Regulator
SAM: Shoot apical meristem
SAUR: Small auxin up RNA
SLY2: Sleepy2
STM: Shoot Meristemless
TCP: Teosinte branched1, Cycloidea, Proliferating cell nuclear antigen factor
TF: Transcriptional Factor
TIBA: 2,3,5-triiodobenzoic acid
TIR1/AFB: F-box Transport Inhibitor Response 1/Auxin Signaling F-box Protein
TPS11: Trehalose-6-Phosphate Synthase 11
Tre6P: Trehalose-6-Phosphate
UGT: Uridine Diphosphate Glycosyltransferase
WOX: WUSCHEL-related homeobox
WUS: WUSCHEL
