# Supplementary material for: Auxin and cytokinin coordinate the dormancy and outgrowth of axillary bud in strawberry runner
Source: BMC Plant Biol. 2019 Nov 29;19:528. doi: 10.1186/s12870-019-2151-x (PMC6884756; doi:10.1186/s12870-019-2151-x)
Supplement: Supplementary file 1 — Additional file 1: Figure S1. Correlation analysis. The value r 2 close to 1 means a stronger correlation between two samples. [file 12870_2019_2151_MOESM1_ESM.pdf]

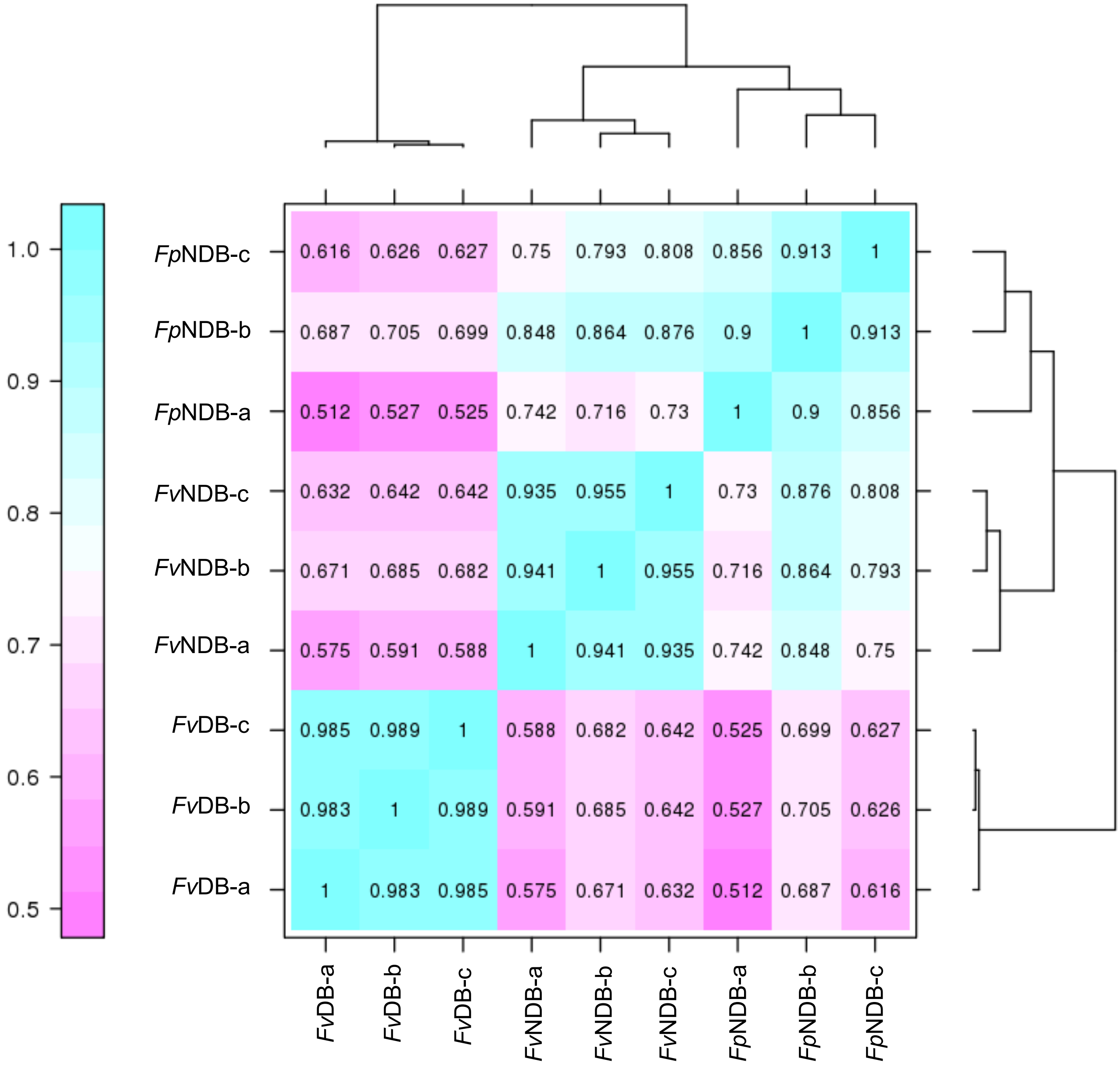

Figure S1. Correlation analysis. The value r^2 close to 1 means a stronger correlation between two samples.
