# Supplementary material for: Auxin and cytokinin coordinate the dormancy and outgrowth of axillary bud in strawberry runner
Source: BMC Plant Biol. 2019 Nov 29;19:528. doi: 10.1186/s12870-019-2151-x (PMC6884756; doi:10.1186/s12870-019-2151-x)
Supplement: Supplementary file 2 — Additional file 2: Figure S2. Volcano plot of different expression analysis. (A) volcano plot of DEGs between FvDB and FvNDB. (B) volcano plot of DEGs between FvDB and FpNDB. (C) volcano plot of DEGs between FvNDB and FpNDB. Differential expression analysis was performed using the DESeq2-R package with following criteria: FDR < 0.01 and Log2FC ≥ 1. [file 12870_2019_2151_MOESM2_ESM.pdf]

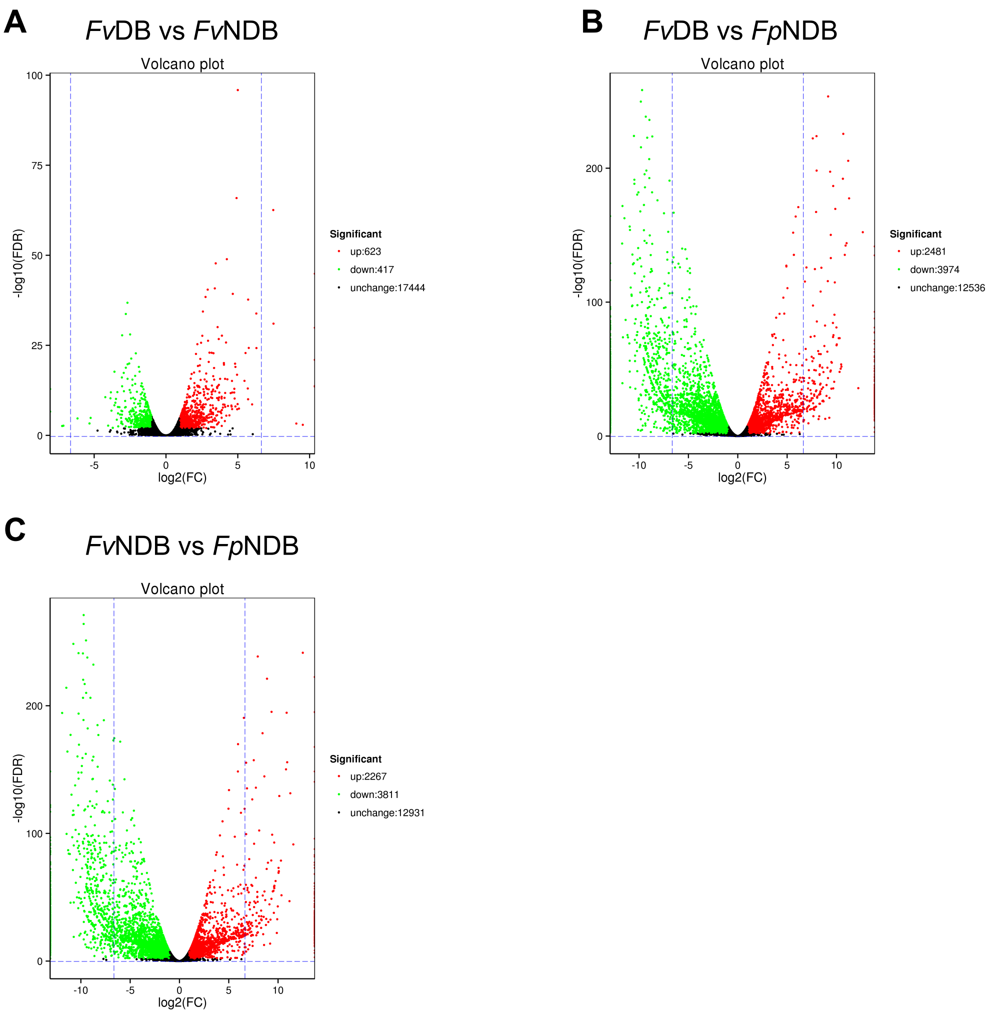

Figure S2. Volcano plot of different expression analysis. (A) volcano plot of DEGs between *Fv*DB and *Fv*NDB. (B) volcano plot of DEGs between *Fv*DB and *Fp*NDB. (C) volcano plot of DEGs between *Fv*NDB and *Fp*NDB. Differential expression analysis was performed using the DESeq2-R package with following criteria: False Discovery Rate (FDR) < 0.01 and Log2FC≥1.
