# Supplementary material for: Auxin and cytokinin coordinate the dormancy and outgrowth of axillary bud in strawberry runner
Source: BMC Plant Biol. 2019 Nov 29;19:528. doi: 10.1186/s12870-019-2151-x (PMC6884756; doi:10.1186/s12870-019-2151-x)
Supplement: Supplementary file 3 — Additional file 3: Figure S3. IAA and Zeatin content were detected by HPLC-MS. The representative chromatograms showed LC separation and MS detection with ESI mode of 13 C 6 -IAA (A) and 15 N 4 -trans Zeatin (C). The standard curves of 13 C 6 -IAA (B) and 15 N 4 -trans Zeatin (D) were made by 5 concentration gradients. [file 12870_2019_2151_MOESM3_ESM.pdf]

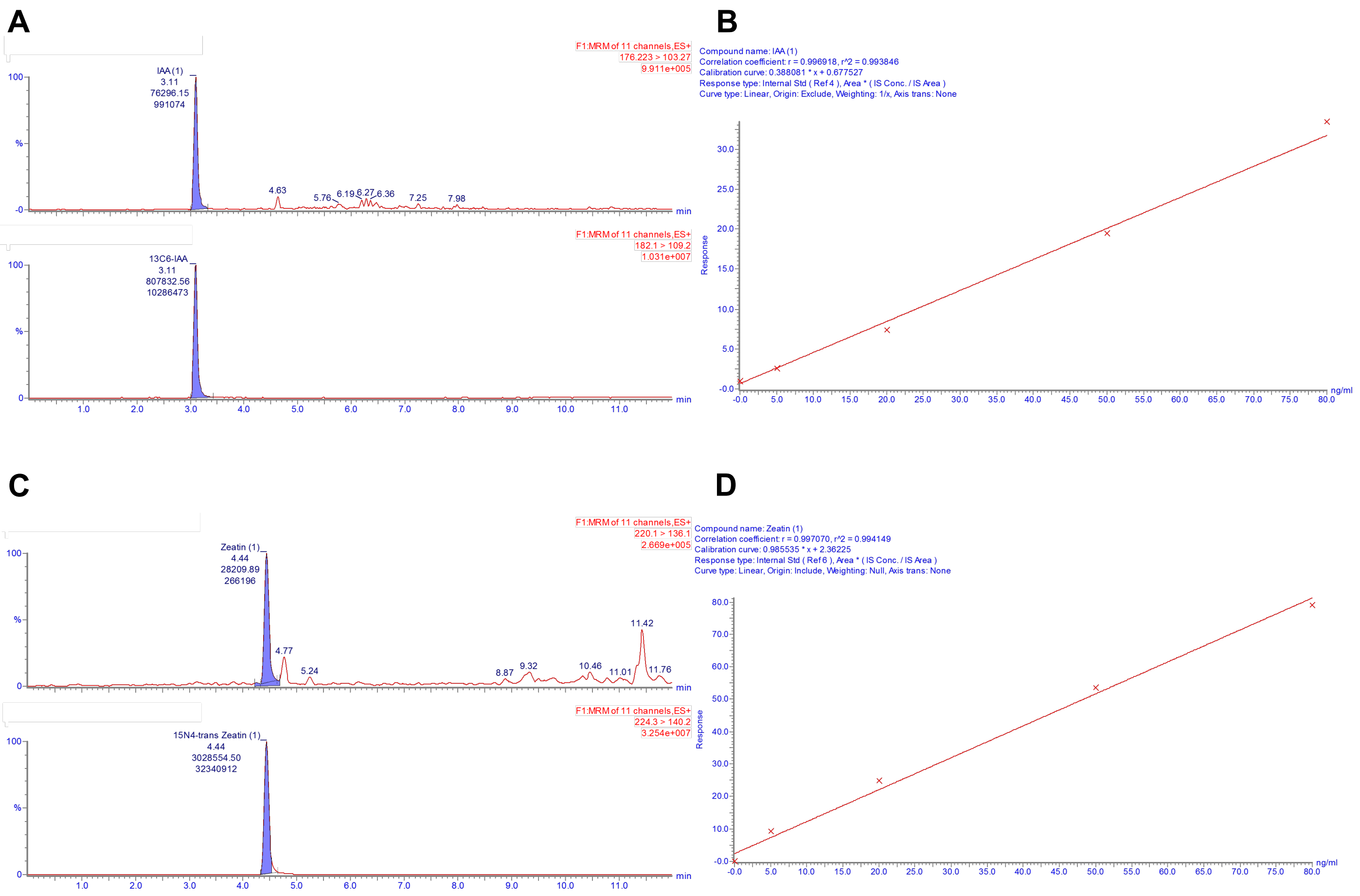

Figure S3. IAA and Zeatin content were detected by HPLC-MS. The representative chromatograms showed LC separation and MS detection with ESI mode of $^{13}C_6$-IAA (A) and $^{15}N_4$-trans Zeatin (C). The standard curves of $^{13}C_6$-IAA (B) and $^{15}N_4$-trans Zeatin (D) were made by 5 concentration gradients.
