# Supplementary material for: Auxin and cytokinin coordinate the dormancy and outgrowth of axillary bud in strawberry runner
Source: BMC Plant Biol. 2019 Nov 29;19:528. doi: 10.1186/s12870-019-2151-x (PMC6884756; doi:10.1186/s12870-019-2151-x)
Supplement: Supplementary file 4 — Additional file 4: Figure S4. (A) The relative expression level of Strigolactone related genes. Error bar indicates the standard deviation obtained from three biological replicates. P-values were determined by two-tailed Student’s t-test *p < 0.05. (B) Heatmap of GA, ABA and sugar metabolism and signaling related genes in the 439 core DEGs. Heatmaps represent transformed genes by the single gradient method. White color shows the lowest value in the heatmap. in contrast, red color shows the highest value in the heatmap. (C) The relative expression level of GA, ABA and sugar metabolism and signaling related genes. Error bar indicates the standard deviation obtained from three biological replicates. P-values were determined by two-tailed Student’s t-test *p < 0.05. [file 12870_2019_2151_MOESM4_ESM.pdf]

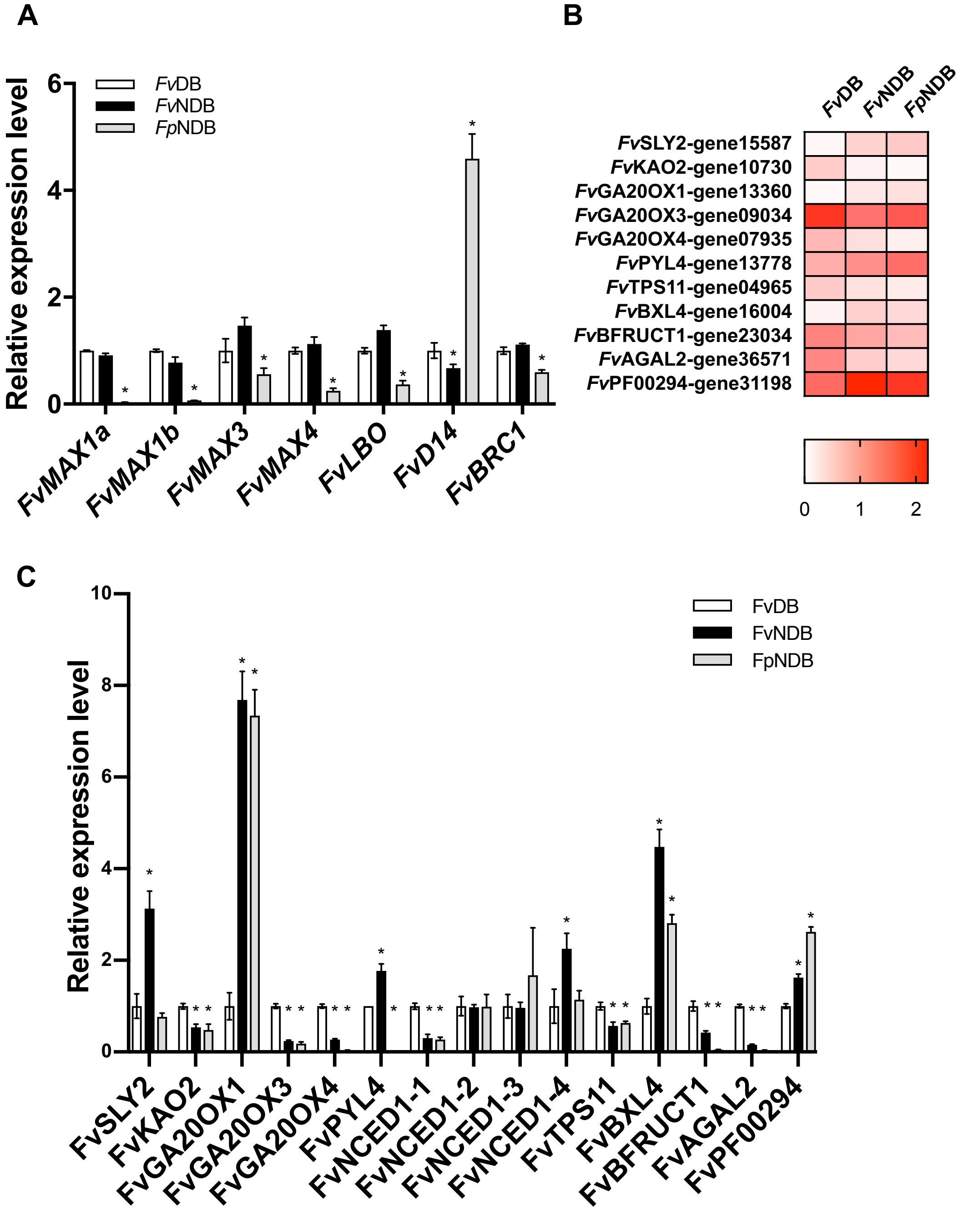

Figure S4. (A)The relative expression level of Strigolactone related genes. Error bar indicates the standard deviation obtained from three biological replicates. P-values were determined by two-tailed Student's t-test *$p<0.05$. (B) Heatmap of GA, ABA and sugar metabolism and signaling related genes in the 439 core DEGs. Heatmaps represent transformed genes by the single gradient method. White color shows the lowest value in the heatmap. in contrast, red color shows the highest value in the heatmap. (C)The relative expression level of GA, ABA and sugar metabolism and signaling related genes. Error bar indicates the standard deviation obtained from three biological replicates. P-values were determined by two-tailed Student's t-test *$p<0.05$.
