# Supplementary material for: Auxin and cytokinin coordinate the dormancy and outgrowth of axillary bud in strawberry runner
Source: BMC Plant Biol. 2019 Nov 29;19:528. doi: 10.1186/s12870-019-2151-x (PMC6884756; doi:10.1186/s12870-019-2151-x)
Supplement: Supplementary file 5 — Additional file 5: Figure S5. Replicates of DR5:GUS straining pictures. 6-BA treated FvDB have two different development types after release from dormancy. One is bud turnover, another is bud outgrowth. SAM: Shoot apical meristem; AM: Axillary meristem; VB: Vascular bundles; LF: Leaf; LP: Leaf primordium; RM: Root meristem [file 12870_2019_2151_MOESM5_ESM.pdf]

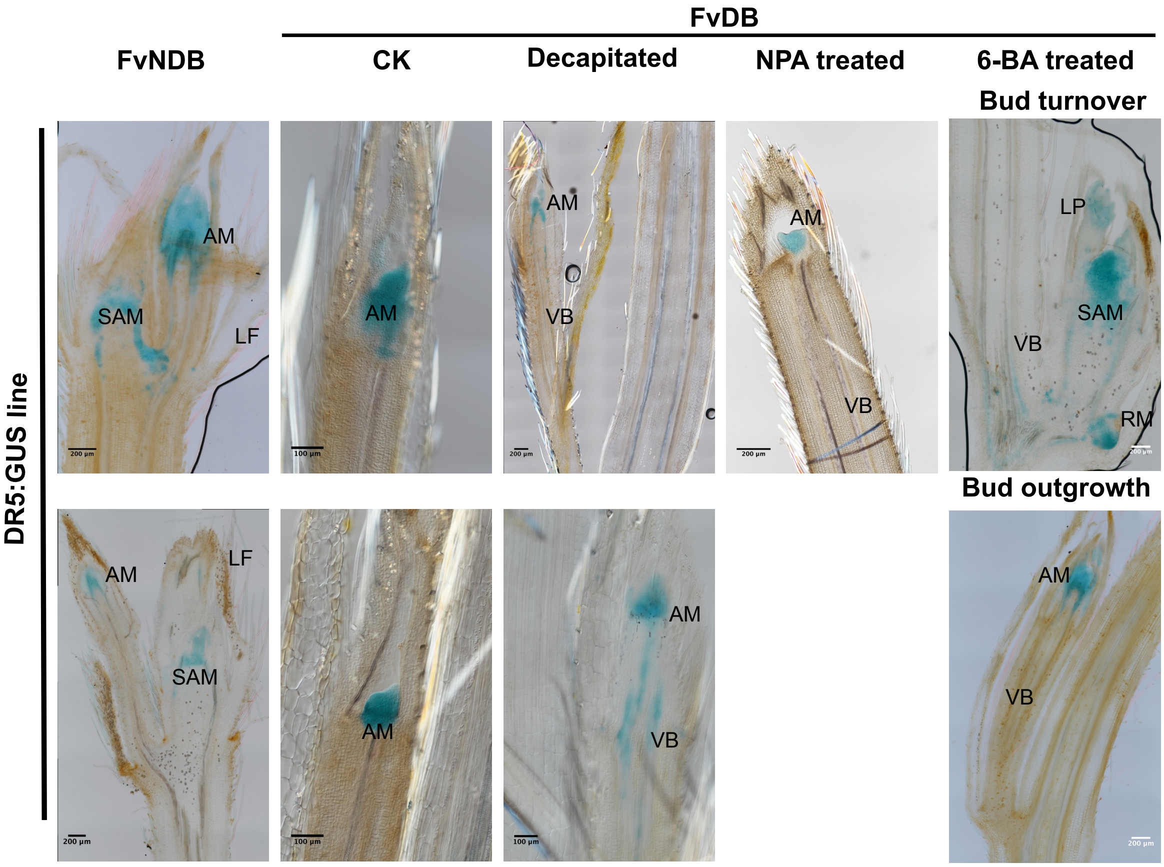

Figure S5. Replicates of DR5:GUS straining pictures. 6-BA treated FvDB have two different development types after release from dormancy. One is bud turnover, another is bud outgrowth. SAM: Shoot apical meristem; AM: Axillary meristem; VB: Vascular bundles; LF: Leaf; LP: Leaf primordium; RM: Root meristem.
