# Supplementary material for: Auxin and cytokinin coordinate the dormancy and outgrowth of axillary bud in strawberry runner
Source: BMC Plant Biol. 2019 Nov 29;19:528. doi: 10.1186/s12870-019-2151-x (PMC6884756; doi:10.1186/s12870-019-2151-x)
Supplement: Supplementary file 6 — Additional file 6: Table S1. Runner pattern in different species of Fragaria. Table S2. Summary of transcriptome sequencing [file 12870_2019_2151_MOESM6_ESM.docx]

**Table S1.** Runner pattern in different species of *Fragaria.*

| **Species** | **runner pattern** |
| --- | --- |
| *F. nipponica* (2n=14) | Type I |
| *F. pentaphylla* (2n=14) | Type I |
| *F. viridis* (2n=14) | Type I |
| *F. moupinensis* (2n=28) | Type I |
| *F. corymbosa* (2n=28) | Type I |
| *F. vesca* (2n=14) | Type II |
| *F. mandschurica* (2n=14) | Type II |
| *F. orientalis* (2n=28) | Type II |
| *F.× bringhurstii* (2n=35) | Type II |
| *F. moschata* (2n=42) | Type II |
| *F. chiloensis* (2n=56) | Type II |
| *F. virginiana* (2n=56) | Type II |
| *F. ×ananassa* (2n=56) | Type II |

Type I: All buds develop to daughter plants except for the first dormant bud (monopodial type); Type II: Only the non-dormant buds develop to daughter plants (sympodial type).

**Table S2.** Summary of transcriptome sequencing.

| **ID** | **Total Reads** | **Mapped Reads** | **Mapped rates** | **Clean bases** | **GC Content** | **%≥Q30** |
| --- | --- | --- | --- | --- | --- | --- |
| *Fv*DB-a | 48558494 | 45569188 | 93.84% | 6.733Gbp | 46.50% | 94.34% |
| *Fv*DB-b | 44368068 | 41561695 | 93.67% | 6.155Gbp | 46.67% | 94.30% |
| *Fv*DB-c | 46883424 | 43823209 | 93.47% | 6.491Gbp | 46.73% | 94.46% |
| *Fv*NDB-a | 43218452 | 40500848 | 93.71% | 6.004Gbp | 46.43% | 94.41% |
| *Fv*NDB-b | 45798538 | 43023279 | 93.94% | 6.351Gbp | 46.66% | 94.46% |
| *Fv*NDB-c | 45277346 | 41974575 | 92.71% | 6.294Gbp | 46.82% | 94.41% |
| *Fp*NDB-a | 44808672 | 33449604 | 74.65% | 6.222Gbp | 47.57% | 94.32% |
| *Fp*NDB-b | 48398924 | 36732366 | 75.90% | 6.725Gbp | 47.61% | 94.38% |
| *Fp*NDB-c | 44455118 | 33214133 | 74.71% | 6.167Gbp | 47.34% | 94.49% |
